# Antispasmodic and analgesic strategies for renal colic in pregnancy: a comprehensive analysis

**DOI:** 10.3389/fphar.2026.1758430

**Published:** 2026-10-01

**Authors:** Bo Lv, Yan Chen, Peiying Huang, Man Liu, Li Chen

**Affiliations:** 1 Guizhou University of Traditional Chinese Medicine, Guiyang, China; 2 First Affiliated Hospital of Guizhou University of Traditional Chinese Medicine, Guiyang, China; 3 School of Basic Medical Science of Guangzhou University of Chinese Medicine, Guangzhou, China; 4 Guangdong Provincial Second Hospital of Traditional Chinese Medicine, Guangdong Provincial Engineering Technology Research Institute of Traditional Chinese Medicine, The Fifth Clinical College of Guangzhou University of Chinese Medicine, Guangdong Provincial Key Laboratory of Research and Development in Traditional Chinese Medicine, Guangzhou, China; 5 Second Affiliated Hospital of Guizhou University of Traditional Chinese Medicine, Guiyang, China; 6 Emergency Department of Guangdong Provincial Hospital of Traditional Chinese Medicine, Guangzhou, China

**Keywords:** anisodamine, phloroglucinol, pregnancy, progesterone, renal colic

## Abstract

**Background:**

Analgesia remains the paramount priority in the clinical management of gestational renal colic, yet established protocols are frequently constrained by contraindications. While antispasmodic intervention emerges as a viable alternative, the definitive pharmacological choice remains elusive. Here, we evaluated the effectiveness and safety of commonly used antispasmodic and analgesic strategies for pregnancy-related renal colic using network and pairwise meta-analyses.

**Method:**

A literature review was undertaken utilizing three English-language databases and four Chinese-language databases, encompassing publications up to 1 January 2025. Randomized controlled trials (RCTs) that satisfied the inclusion criteria were selected for analysis. The Cochrane Risk-of-Bias Tool was employed to evaluate the risk of bias. Statistical analyses were performed, encompassing intervention comparison and ranking, assessment of consistency and heterogeneity, evaluation of publication bias, as well as meta-regression, subgroup, and sensitivity analyses.

**Results:**

The study included ten RCTs with 936 patients. Four dimensions for the RCTs had a low risk of bias, with one dimension having “some concerns.” In network meta-analyses, phloroglucinol was most effective in relieving pain within 1 h, followed by progesterone, while anisodamine was least effective. Phloroglucinol caused the least side effects like nausea and dizziness compared to progesterone and anisodamine. In pairwise meta-analyses, phloroglucinol had a lower 6-h pain recurrence rate and fewer side effects like blurred vision and palpitations than anisodamine. Variations in phloroglucinol dosage contributed to heterogeneity, but subgroup and sensitivity analyses confirmed result stability.

**Conclusion:**

Phloroglucinol is the preferred treatment for renal colic pain during pregnancy due to its efficacy and safety. It can be combined with progesterone, but anisodamine is not advised. Further high-quality research is necessary to confirm this finding.

**Clinical Trial Registration:**

https://www.crd.york.ac.uk/PROSPERO/view/CRD42022377502.

## Introduction

During pregnancy, renal colic represents the most prevalent non-obstetric cause of abdominal pain and subsequent hospitalization, particularly during the second and third trimesters (Rosenberg et al., 2011). It poses a significant risk factor for various pregnancy complications, including gestational hypertension syndrome, increased rates of low-birth-weight infants, premature rupture of membranes, spontaneous abortion, and preterm birth (Ordon et al., 2020; Kirubarajan et al., 2022).

The occurrence of renal colic during pregnancy is primarily attributed to two distinct mechanisms. The first involves mechanical pressure exerted by the expanding uterus on the urinary tract. The inherent dextrorotation of the uterus, combined with vascular congestion within the uteroplacental circulation, renders the right ureter particularly susceptible to compression (Edyedu et al., 2025). This mechanical obstruction leads to physiological hydroureteronephrosis and renal colic, which affect predominantly the right kidney and ureter during the second and third trimesters (Travassos et al., 2009). The second and most frequent cause of renal colic in pregnant women is nephrolithiasis, with an incidence ranging from 1:125 to 1:2,000 (Lewis et al., 2003; Ordon et al., 2020; Sohlberg et al., 2020). Pregnancy provides favorable physiological conditions for stone formation. Research indicates that during the second trimester, the glomerular filtration rate increases by 40%–65% and renal plasma flow rises by 50%–85% compared to non-pregnant levels (Semins and Matlaga, 2010). These hemodynamic alterations lead to elevated urinary excretion of cholesterol, oxalate, uric acid, and calcium, thereby increasing the risk of lithogenesis (Edyedu et al., 2025). Additionally, increased placental secretion of 1,25-dihydroxycholecalciferol and decreased parathyroid hormone levels result in transient hypercalciuria, which further promotes stone development (Semins et al., 2009). Simultaneously, elevated maternal progesterone levels induce smooth muscle relaxation, diminished peristalsis, and slowed urinary transit, while also causing ureteral dilation, elongation, and tortuosity (Evans and Wollin, 2001; Semins et al., 2009). These changes collectively result in urinary stasis and dilation of the renal pelvis and ureters, creating a microenvironment conducive to stone formation. Once formed, the migration of a calculus within the renal pelvis or ureter obstructs urinary flow, causing a rapid increase in intraluminal pressure proximal to the obstruction and eliciting severe pain (Semins et al., 2009). Furthermore, the calculus acts as a foreign body that directly irritates the ureteral mucosa, triggering intense spasmodic contractions of the ureteral smooth muscle (Rana et al., 2009). Beyond the pain caused by these muscle spasms, the increased intraluminal pressure due to fluid retention further exacerbates the discomfort. It is noteworthy that urinary obstruction leads to stasis, which facilitates bacterial proliferation and predisposes individuals to urinary tract infections (Webb, 2000). The subsequent inflammatory response intensifies local edema and sensitizes nerve endings, thereby amplifying the perception of pain (Bozkurt et al., 2012).

In clinical practice, approximately 25%–30% of patients ultimately necessitate surgical intervention (double J stent placement, nephrostomy, or definitive stone removal) due to persistent symptoms or other complications (Pedro et al., 2016; Dai et al., 2021). Nevertheless, surgical procedures are associated with increased risks, including anesthesia-related complications, infection, and hemorrhage (Somani et al., 2017; Laranjo et al., 2024). According to the current guidelines of the American Urological Association, conservative management is advocated as the first-line treatment for stable renal colic during pregnancy (Assimos et al., 2016). These conservative strategies encompass fluid replacement, analgesia, antiemetic therapy, and vigilant monitoring, with pain management being paramount, as severe pain elevates the risk of miscarriage, and persistent pain serves as an indication for surgical intervention (Dai et al., 2021; Juliebø-Jones et al., 2022). Under conservative management, spontaneous passage of stones is observed in 23%–84% of patients (Dai et al., 2021).

Compared with non-pregnant patients, analgesic options for the management of renal colic during pregnancy are limited (Ho et al., 2025). Paracetamol is currently the internationally recommended first-line analgesic (D'Antonio et al., 2026). Although the suspected association between paracetamol and autism spectrum disorder, attention-deficit hyperactivity disorder, or intellectual disability is now considered unsupported by evidence, some studies suggest that its administration may increase the risks of cryptorchidism and asthma in children (Burdan et al., 2012; D'Antonio et al., 2026). Maternal complications such as pre-eclampsia, preterm birth, venous thromboembolism, and pulmonary embolism are also noted as potential risks (Burdan et al., 2012). Other non-selective cyclooxygenase inhibitors, specifically non-steroidal anti-inflammatory drugs (NSAIDs), are associated with intrauterine growth restriction, constriction of the ductus arteriosus with secondary persistent pulmonary hypertension, and oligohydramnios due to reduced fetal renal perfusion (Burdan et al., 2012). NSAIDs are further linked to the prolongation of pregnancy and an increased incidence of neonatal intracranial hemorrhage (Burdan et al., 2012). Specifically, high-dose acetylsalicylic acid presents a significant risk of miscarriage, stillbirth, and various congenital malformations (Burdan et al., 2012). Codeine is contraindicated in the early stages of pregnancy owing to its teratogenic effects (Broussard et al., 2011; Thakur et al., 2020). Prolonged use of high-dose morphine may result in fetal drug dependence and is associated with intrauterine growth restriction and preterm birth (Juliebø-Jones et al., 2022). While some narcotics are generally deemed safe, they may still be linked to neonatal withdrawal syndrome (Thakur et al., 2020). Alpha-adrenoreceptor blockers, such as tamsulosin, are occasionally used by a small number of clinicians; however, robust clinical evidence supporting their efficacy remains insufficient, and the potential risks associated with their use have not been fully elucidated (Thakur et al., 2020; Deng et al., 2024). Consequently, selecting an appropriate analgesic regimen for pregnant patients presents a clinical challenge.

In cases where conventional analgesics are contraindicated or provide insufficient relief, medications that alleviate pain through the relaxation of smooth muscle spasms serve as alternative options. This clinical preference is validated by a survey involving 110 urologists which examines the selection of pain management strategies for pregnancy-related renal colic. Within this clinical context, phloroglucinol, progesterone, and anisodamine are frequently employed to address renal colic in pregnant patients (Deng et al., 2024). Nonetheless, within a safe range, there are still differences in efficacy and side effects among them. There is a lack of systematic evaluation regarding which of these medications offers the optimal analgesic efficacy with minimal side effects in this population. Consequently, we undertake a meta-analysis to systematically assess the benefits and drawbacks of phloroglucinol, progesterone, and anisodamine in treating renal colic among pregnant women.

## Methods

Our research was conducted in accordance with the PRISMA extensions statement and has been registered on PROSPERO (CRD42022377502). The PRISMA checklist for this study can be viewed in Supplementary File S1.

### Search strategy

We conducted a search using the keywords “phloroglucinol”, “progesterone”, “anisodamine”, “renal colic”, “pregnan*”, and “randomized controlled trial” in PubMed, Embase, Cochrane Library, China National Knowledge Infrastructure, Wanfang Database, Weipu Journal Database, and Chinese Biomedical Literature Database. The search period was limited from the establishment of the databases to 1 January 2025. The detailed search terms for each database can be found in Supplementary File S2.

### Study selection

The study population was restricted to pregnant women experiencing renal colic, with no limitations imposed on age, geographic region, or ethnicity. Throughout the study duration, participants did not undergo any surgical interventions. Within the framework of conservative management for renal colic, each patient group was assigned a single analgesic agent from the following options: phloroglucinol, progesterone, or anisodamine. The outcome measures included at least one of the following: 1-h pain relief rate, 6-h pain recurrence rate, and the incidence of potential drug-related side effects. The study was classified as a randomized controlled trial (RCT). Exclusion criteria encompassed studies involving participants with mental disorders, severe underlying medical conditions, or concurrent pain-related complications. Additionally, studies involving participants engaged in other research projects or employing flawed randomization methods were excluded. The literature screening process was conducted independently by two researchers, and in instances of uncertainty, a third party was consulted to reach a definitive decision.

### Data extraction

For each included study, we extracted the following information: publication year, researcher name, randomization method, demographic characteristics, comparison of baseline data between groups, prescription of analgesics, pain measurement methods, reported outcome situation, and research funding details. Data extraction is conducted independently by two researchers. Upon completion, the results are compared, and any discrepancies are resolved through mutual discussion or through the intervention of a third researcher who facilitates the final determination.

### Quality assessment

We employed Version 2 of the Cochrane Risk-of-Bias tool (RoB 2) to perform a quality assessment across five dimensions: Randomization process, Deviations from intended interventions, Missing outcome data, Measurement of the outcome, and Selection of the reported result. The final quality level of each study was determined by the lowest rating among these sub-items. This assessment was independently conducted by two researchers, with a third party resolving any disagreements.

### Data analysis

In this study, a network meta-analysis was conducted within a Bayesian framework to evaluate outcomes associated with three interventions. The model was fitted using four Markov chains, with a total of 200,000 iterations and an initial burn-in period of 10,000 iterations. Network plots were utilized to visualize direct comparisons among interventions. Model selection was guided by the deviance information criterion (DIC), which was employed to compare competing models, including consistency versus inconsistency models and random-effects versus fixed-effects models. Models with lower DIC values were considered to exhibit superior fit and stability (Béliveau et al., 2019). Furthermore, the DIC was used to assess the consistency assumption, with a difference of less than 5 between consistent and inconsistent models indicating adherence to this assumption (Béliveau et al., 2019).

The synthesis of direct and indirect evidence was illustrated through a league heat plot, while the ranking of interventions was evaluated using SUCRA plots (Surface Under the Cumulative Ranking). Heterogeneity was assessed at both the pairwise level, utilizing the *I* ^*2*^ statistic, and the overall level, employing the Global *I* ^*2*^ statistic. For outcomes involving only two interventions, pairwise meta-analysis was performed: a random-effects model was employed when *I* ^*2*^ > 50%, whereas a fixed-effects model was used when *I* ^*2*^ < 50%, with *I* ^*2*^ serving as a measure of heterogeneity. Due to limitations in the original literature, such as incomplete reporting of critical variables like stone size and gestational age, some data were either unavailable or inconsistent across studies, thereby impeding standardized analysis. Significant variables identified through these regressions informed subsequent subgroup analyses. Additionally, sensitivity analyses were conducted to further evaluate robustness and heterogeneity. Publication bias was assessed using funnel plots. Network meta-analyses were conducted employing the ‘gemtc’ and ‘BUGSnet’ packages in R, whereas pairwise meta-analyses were performed using the ‘meta’ package. Statistical significance was established based on confidence intervals that did not encompass the null value.

## Result

### Study characteristics

Utilizing the predefined search strategy, a total of 77 literature entries were initially identified. Following both software-assisted and manual de-duplication processes, along with a rigorous evaluation against predetermined criteria, 10 studies were ultimately included in this meta-analysis. The flow chart detailing the study selection process is provided in Supplementary File S3.

This analysis included ten studies with a total of 936 pregnant participants. All the RCTs were two-arm studies. Eight of these studies compared the clinical outcomes of phloroglucinol and anisodamine, while two compared phloroglucinol and progesterone. The network meta-analysis assessed clinical outcomes such as 1-h pain relief rate and the incidence of nausea, dry mouth, flushing, urinary difficulties, dizziness, and headaches. Some outcomes, like the 6-h pain recurrence rate, incidence of blurred vision, and palpitations, were analyzed using pairwise meta-analysis, focusing only on phloroglucinol and anisodamine. Table 1 provides detailed information about each study, and Figure 1 illustrates the direct comparisons of interventions for each outcome in the network meta-analysis.

**TABLE 1:** Detailed information for the included studies.

Study	Sample size (E/C)	Age (year, E/C)	Pregnancy duration (week, E/C)	Intervention in the experimental group	Intervention in the control group	Outcomes
Wang (2015)	25/25	25–30, 28.6 ± 0.7/25–30, 28.1 ± 1.3	18–35, 24.9 ± 1.2/18–32, 23.6 ± 1.5	Phloroglucinol 60 mg intravenous drip	Anisodamine 10 mg intravenous drip	①
Cai (2013)	30/30	-	-	Phloroglucinol 60 mg intravenous drip	Anisodamine 10 mg intravenous drip	① ④ ⑥ ⑦ ⑧ ⑨
Guan (2011)	21/19	20–38	-	Phloroglucinol 80 mg intravenous drip	Anisodamine 20 mg intravenous drip	① ②
Cai et al. (2014)	30/30	18–42, 29.75 ± 2.25	30.57 ± 3.38	Phloroglucinol 80 mg intravenous drip	Anisodamine 10 mg intravenous drip	① ② ④ ⑤ ⑦ ⑧ ⑨
Liang (2011)	20/20	20.5–37.8	-	Phloroglucinol 80 mg intravenous drip	Anisodamine 10 mg intravenous drip	① ② ⑥ ⑦ ⑧ ⑨
Qiu and Luo (2011)	38/26	20–31, 28.25 ± 2.24	24.13 ± 6.18	Phloroglucinol 40 mg intramuscular injection	Anisodamine 10 mg intramuscular injection	① ③ ④ ⑥ ⑦ ⑨
Hu (2011)	30/30	19–38	16–34	Phloroglucinol 80 mg intravenous drip	Anisodamine 10 mg intravenous drip	① ② ⑥ ⑦ ⑧ ⑨
Huang (2020)	46/46	18–40, 32.37 ± 3.25/20–40, 33.25 ± 3.46	-	Phloroglucinol 40 mg intravenous drip	Progesterone 20 mg intravenous drip	①
Zhai et al. (2018)	155/155	21–43, 25.46 ± 6.22/22–45, 25.04 ± 7.03	15–40, 30.55 ± 10.73/15–40, 29.32 ± 11.45	Phloroglucinol 40 mg intravenous drip	Progesterone 40 mg intravenous drip	①
Ou (2014)	80/80	23–47, 36.2 ± 7.6/22–45, 38.4 ± 5.7	12–35, 17.2 ± 5.3/10–37, 18.4 ± 5.7	Phloroglucinol 40–60 mg intravenous drip	Anisodamine 20 mg intravenous drip	① ③ ④ ⑤ ⑧

Outcomes reported in each study: ① One-hour Pain Relief Rate, ② Six-hour Pain Recurrence Rate, ③ Nausea Incidence,④ Dry Mouth Incidence, ⑤ Incidence of Hot Flashes or Flushing, ⑥ Dysuria Incidence, ⑦ Incidence of Headache and Dizziness, ⑧ Blurred Vision Incidence, ⑨ Palpitations Incidence.

**FIGURE 1:**
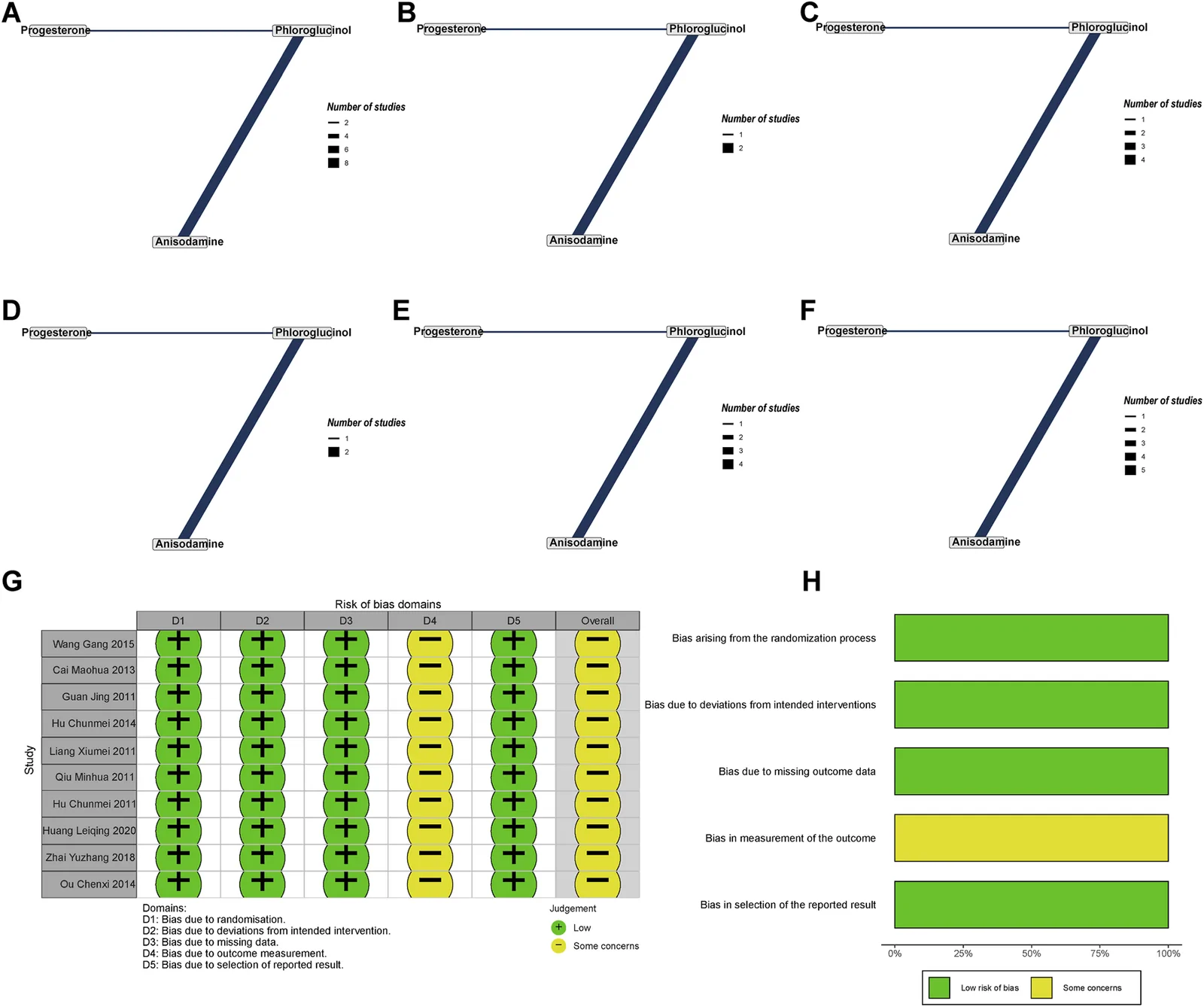
Network plots and quality assessment of the included studies. **(A)** network plots for One-hour Pain Relief Rate; **(B)** network plots for Nausea Incidence; **(C)** network plots for Dry Mouth Incidence; **(D)** network plots for Incidence of Hot Flashes or Flushing; **(E)** network plots for Dysuria Incidence; **(F)** network plots for Incidence of Headache and Dizziness; **(G)** Quality assessment of the individual studies; **(H)** Overall quality assessment of the included studies.

### Methodological quality and risk of bias results

All outcomes in this study were based on subjective patient reports. Consequently, we evaluated the risk of bias for all studies using a unified risk of bias assessment tool.

The included studies implemented randomization methods, with two studies explicitly indicating the use of random number tables (Wang, 2015; Huang, 2020). Each study employed sealed envelopes and adhered to the allocation concealment process. Notably, the baseline characteristics were balanced across all study groups, leading to the randomization process being assessed as low risk.

Blinding was absent in all studies. However, given that the three analgesics examined in this study are routinely used for managing renal colic in pregnant women, the likelihood of patients requesting a change in analgesics during acute pain episodes is considered minimal. Importantly, all studies utilized either intention-to-treat (ITT) or modified intention-to-treat (mITT) analysis; as a result, deviations from intended interventions were also rated as having a low risk of bias.

Regarding missing outcome data, the number of participants in each group aligned with the figures reported in the results, with no missing data identified; consequently, this aspect was assessed as low risk.

In assessing measurement of the outcome, it was noted that all outcomes were subjective, and blinding was not implemented, which could have led to outcomes being influenced by patients’ subjective preferences. Nevertheless, patients lacking any vested interest are generally unable to determine which analgesic is more effective, which may mitigate this subjective influence. As a result, this aspect was conservatively rated as having some concern.

Furthermore, all studies utilized ITT or mITT analysis for data analysis. Pain measurement consistently conducted using the Visual Analog Scale (VAS) score, and the statistical methods were uniform across all studies. Consequently, the selection of the reported result was assessed as having a low risk of bias.

Overall, by applying the principle of getting the lowest score, all studies were rated as having some concern (Figure 1).

### Network meta-analysis

Given that models with lower DIC values are generally more stable, the random and consistency models were chosen for further analysis (Figure 2). The DIC value differences between the consistent and inconsistent models across all outcomes were less than 5, thereby confirming the consistency of each model.

**FIGURE 2:**
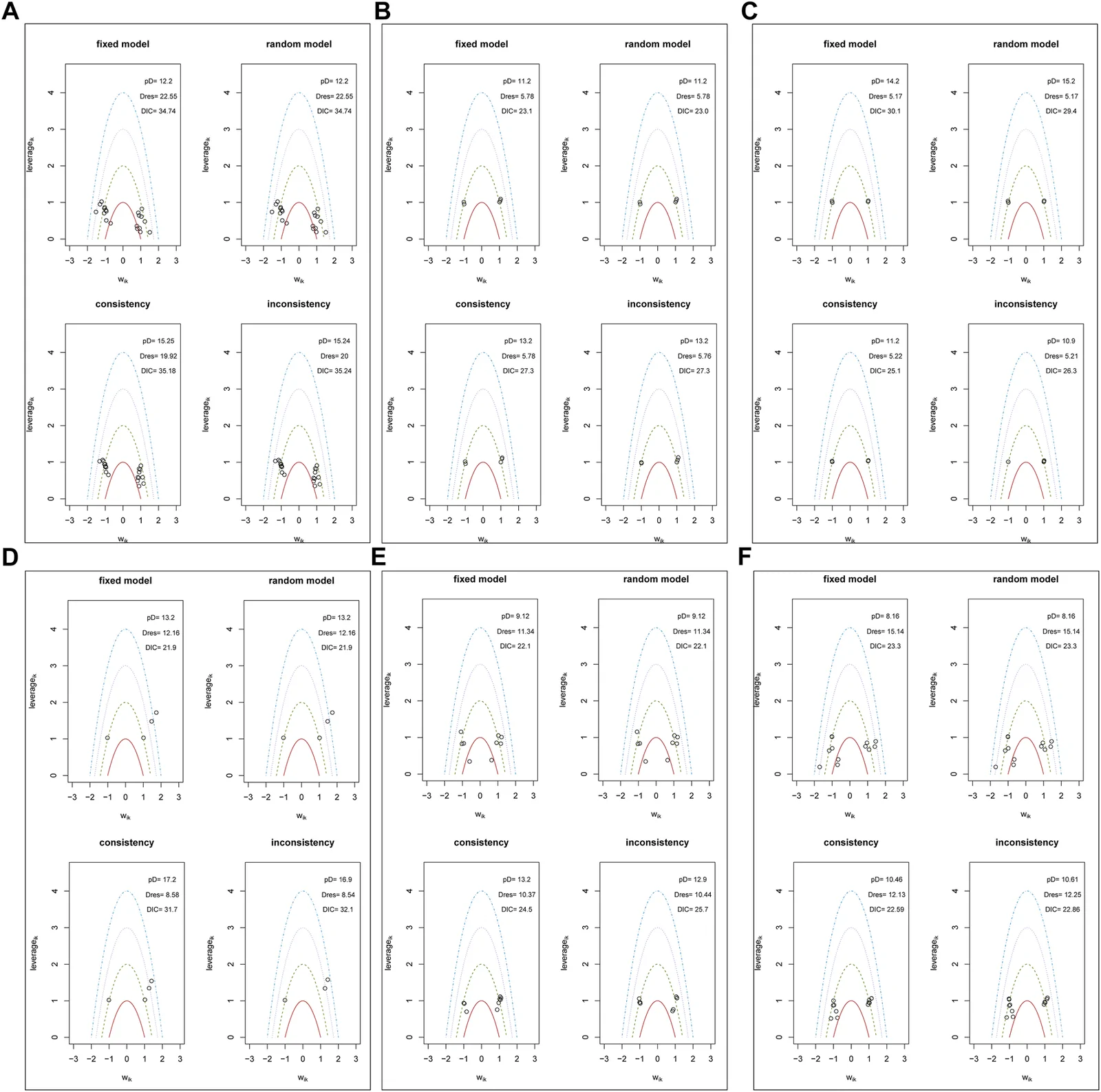
Model fitting results. **(A)** model fitting curve for One-hour Pain Relief Rate; **(B)** model fitting curve for Nausea Incidence; **(C)** model fitting curve for Dry Mouth Incidence; **(D)** model fitting curve for Incidence of Hot Flashes or Flushing; **(E)** model fitting curve for Dysuria Incidence; **(F)** model fitting curve for Incidence of Headache and Dizziness.

### One-hour pain relief rate

The included studies assessed the 1-h pain relief rate (Guan, 2011; Hu, 2011; Liang, 2011; Qiu and Luo, 2011; Cai, 2013; Cai et al., 2014; Ou, 2014; Wang, 2015; Zhai et al., 2018; Huang, 2020). Comparative analysis of interventions demonstrated that phloroglucinol was superior to anisodamine (Figure 3A). According to SUCRA, phloroglucinol ranked highest, followed by progesterone, with anisodamine ranking lowest (Figure 4A). The Global *I* ^*2*^ statistic for the outcome was 24.06%, whereas the *I* ^*2*^ for the comparison between phloroglucinol and progesterone was 72.17% (Figure 5A).

**FIGURE 3:**
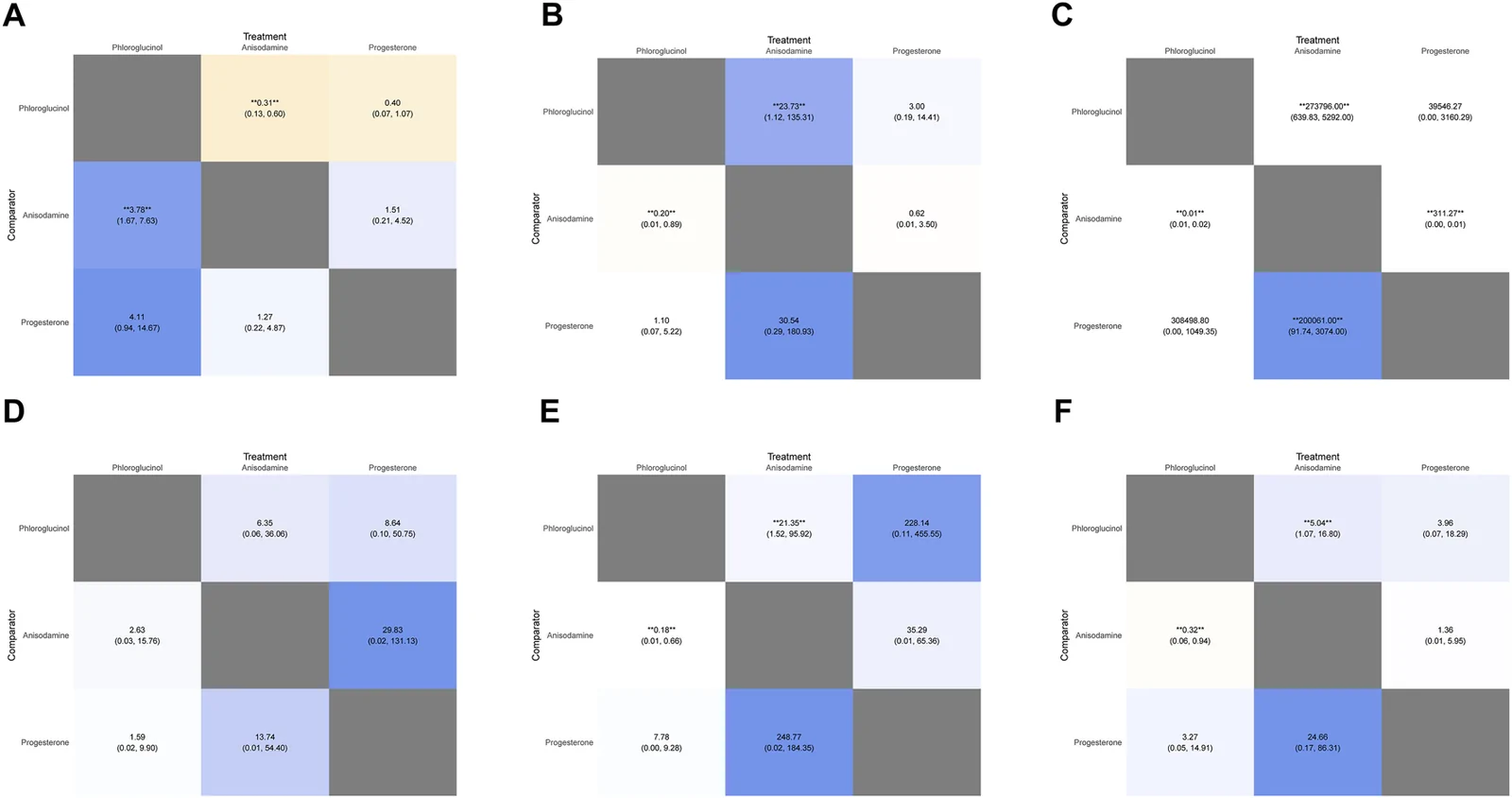
Comparison of effectiveness and safety for phloroglucinol, progesterone and anisodamine. **(A)** league table heatmap for One-hour Pain Relief Rate; **(B)** league table heatmap for Nausea Incidence; **(C)** league table heatmap for Dry Mouth Incidence; **(D)** league table heatmap for Incidence of Hot Flashes or Flushing; **(E)** league table heatmap for Dysuria Incidence; **(F)** league table heatmap for Incidence of Headache and Dizziness.

**FIGURE 4:**
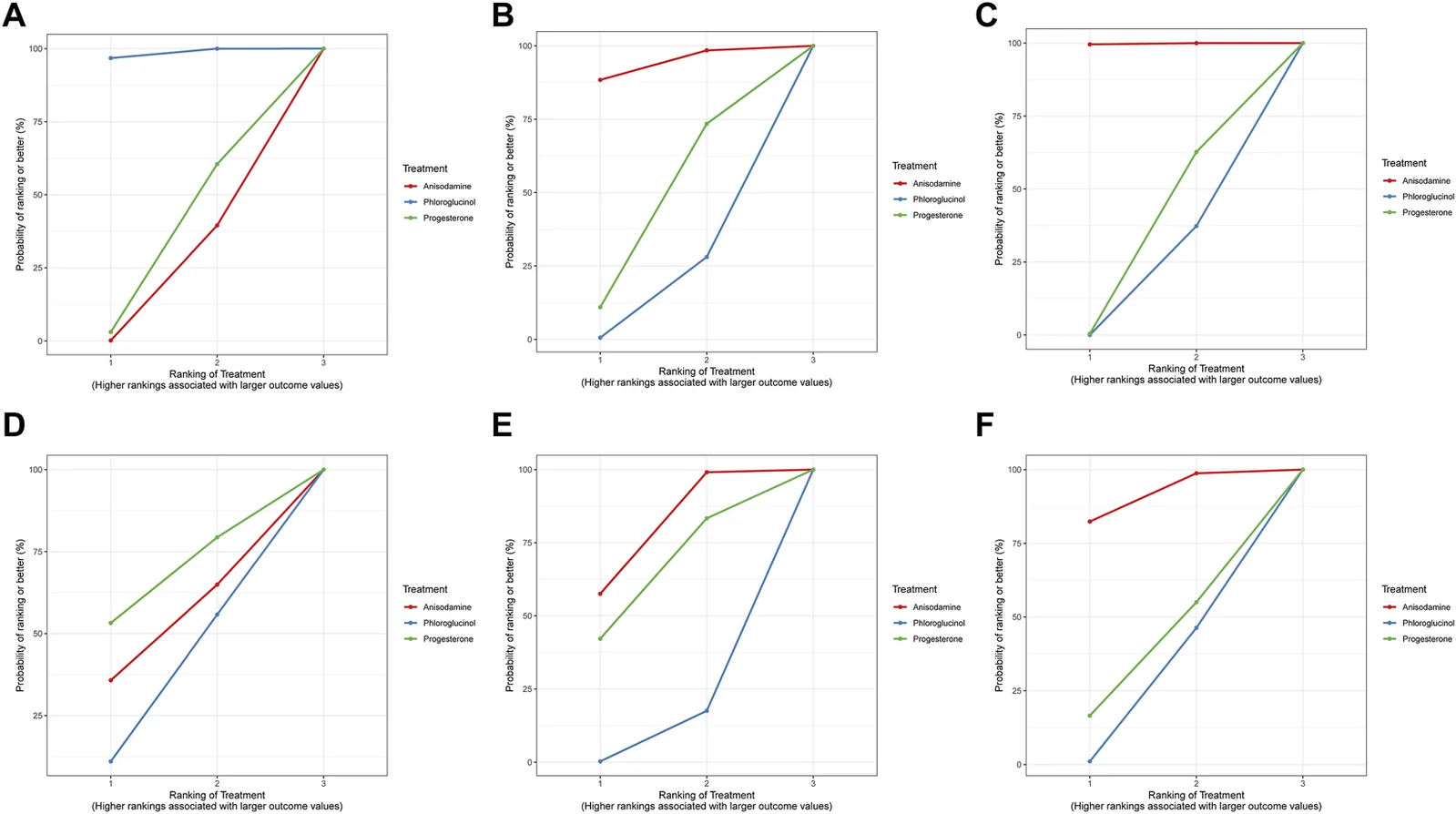
Ranking results based on the Surface Under the Cumulative Ranking (SUCRA). **(A)** SUCRA for One-hour Pain Relief Rate; **(B)** SUCRA for Nausea Incidence; **(C)** SUCRA for Dry Mouth Incidence; **(D)** SUCRA for Incidence of Hot Flashes or Flushing; **(E)** SUCRA for Dysuria Incidence; **(F)** SUCRA for Incidence of Headache and Dizziness.

**FIGURE 5:**
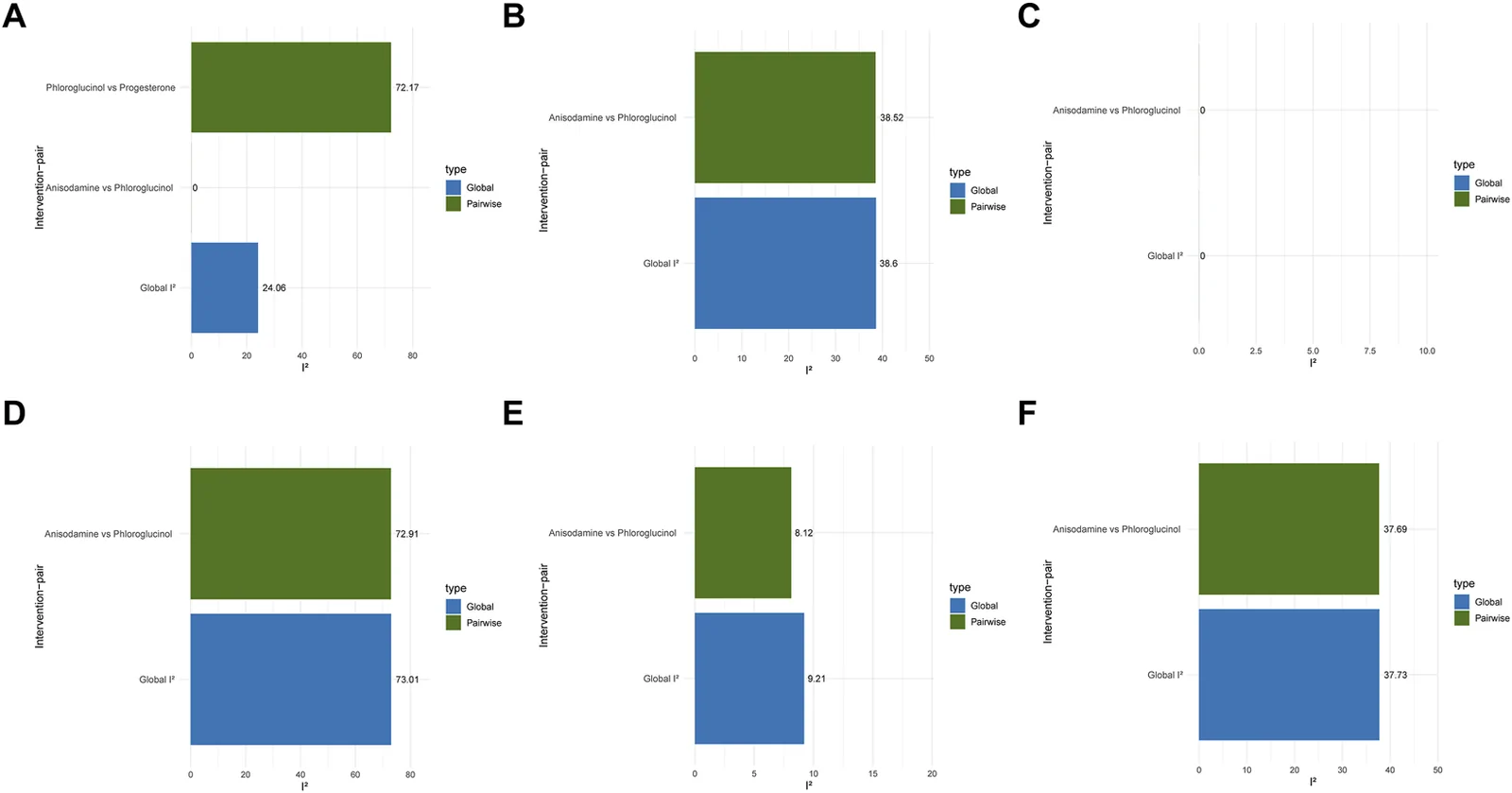
Heterogeneity analysis results. **(A)** heterogeneity analysis results for One-hour Pain Relief Rate; **(B)** heterogeneity analysis results for Nausea Incidence; **(C)** heterogeneity analysis results for Dry Mouth Incidence; **(D)** heterogeneity analysis results for Incidence of Hot Flashes or Flushing; **(E)** heterogeneity analysis results for Dysuria Incidence; **(F)** heterogeneity analysis results for Incidence of Headache and Dizziness.

### Adverse reactions

#### Nausea incidence

Seven studies assessed the incidence of nausea following medication administration (Hu, 2011; Liang, 2011; Qiu and Luo, 2011; Cai, 2013; Cai et al., 2014; Ou, 2014; Zhai et al., 2018). Intergroup comparisons showed that the incidence of nausea was lower after phloroglucinol injection compared to anisodamine (Figure 3B). The SUCRA analysis indicated that anisodamine injection was associated with the highest incidence of nausea, followed by progesterone, with phloroglucinol exhibiting the lowest incidence (Figure 4B). Regarding heterogeneity, the Global *I* ^*2*^ was 38.6%, and the *I* ^*2*^ for the comparison between anisodamine and phloroglucinol was 38.52% (Figure 5B).

#### Dry mouth incidence

Seven studies evaluated the incidence of dry mouth after medication use (Hu, 2011; Liang, 2011; Qiu and Luo, 2011; Cai, 2013; Cai et al., 2014; Ou, 2014; Zhai et al., 2018). Comparative analysis among the interventions demonstrated that the incidence of dry mouth was lower following phloroglucinol injection compared to anisodamine (Figure 3C). SUCRA results indicated that anisodamine had the highest incidence of dry mouth after injection, followed by progesterone, with phloroglucinol being the lowest (Figure 4C). Concerning heterogeneity, both the Global *I* ^*2*^ and the *I* ^*2*^ for the comparison between anisodamine and phloroglucinol were 0.00% (Figure 5C).

#### Incidence of hot flashes or flushing

Seven studies evaluated the incidence of hot flashes or flushing after medication administration (Hu, 2011; Liang, 2011; Qiu and Luo, 2011; Cai, 2013; Cai et al., 2014; Ou, 2014; Zhai et al., 2018). The findings indicated no statistically significant differences among the various interventions (Figure 3D). Nonetheless, SUCRA analysis suggested that progesterone injection was associated with the highest probability of inducing hot flashes or flushing, followed by anisodamine, while phloroglucinol was associated with the lowest probability (Figure 4D). The Global *I* ^*2*^ was 73.01%, and the *I* ^*2*^ for the comparison between phloroglucinol and anisodamine was 72.91% (Figure 5D).

#### Dysuria incidence

Six studies contributed to the evaluation of dysuria incidence following medication administration (Hu, 2011; Liang, 2011; Qiu and Luo, 2011; Cai, 2013; Cai et al., 2014; Zhai et al., 2018). Comparative analysis of interventions demonstrated a lower incidence of dysuria following phloroglucinol injection compared to anisodamine (Figure 3E). The SUCRA-based ranking analysis revealed that anisodamine had the highest probability of causing dysuria, followed by progesterone, with phloroglucinol having the lowest probability (Figure 4E). Regarding heterogeneity, the *I* ^*2*^ statistic for the direct comparison between anisodamine and phloroglucinol was 8.12%, and the Global *I* ^*2*^ was 9.21% (Figure 5E).

#### Incidence of headache and dizziness

Six studies were involved in evaluating the incidence of headache and dizziness following medication administration (Hu, 2011; Liang, 2011; Qiu and Luo, 2011; Cai, 2013; Cai et al., 2014; Zhai et al., 2018). Intervention comparisons showed that the incidence of headache and dizziness after phloroglucinol injection was lower than that after anisodamine (Figure 3F). SUCRA-based ranking analysis indicated that anisodamine had the highest likelihood of causing headache and dizziness, followed by progesterone, with phloroglucinol having the lowest likelihood (Figure 4F). Regarding heterogeneity, the *I* ^*2*^ for the direct comparison between anisodamine and phloroglucinol was 37.69%, and the Global *I* ^*2*^ was 37.73% (Figure 5F).

### Pairwise meta-analysis

#### Six-hour pain recurrence rate

Four studies reported the 6-h pain recurrence rate (Guan, 2011; Hu, 2011; Liang, 2011; Cai et al., 2014). The *I* ^*2*^ value for the model was 0.00%, leading to the selection of a fixed-effect model for analysis. The findings demonstrated that the 6-h pain recurrence rate was lower following phloroglucinol injection compared to anisodamine (Figure 6A).

**FIGURE 6:**
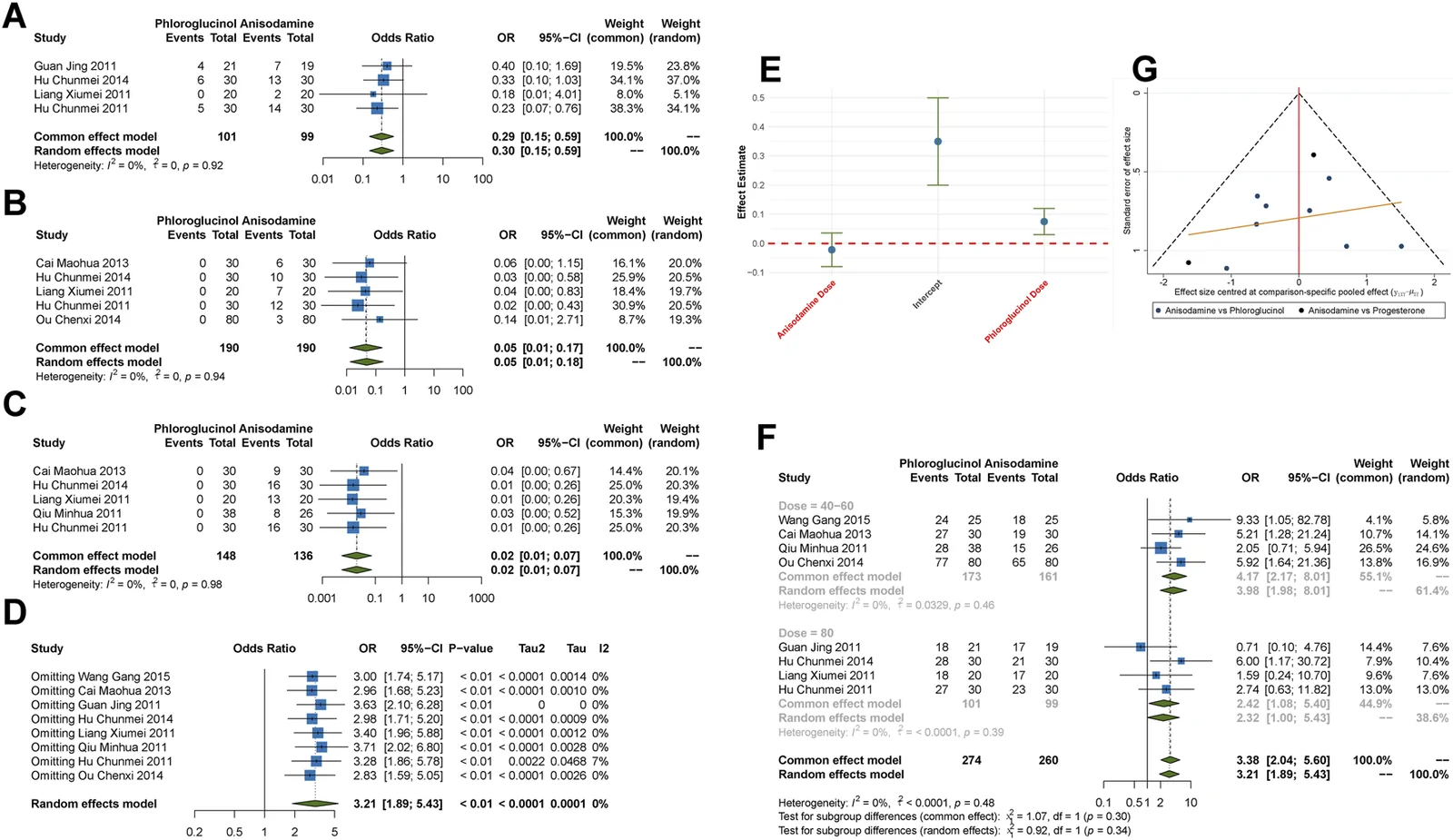
Results of pairwise meta-analysis, meta regression, subgroup analysis, sensitivity analysis, and publication bias. **(A)** the comparison results of the Six-hour Pain Recurrence Rate between phloroglucinol and anisodamine; **(B)** the comparison results of the Incidence of Blurred Vision between phloroglucinol and anisodamine; **(C)** the comparison results of the Incidence of Palpitations between phloroglucinol and anisodamine; **(D)** the results of sensitivity analysis; **(E)** the results of meta regression; **(F)** the results of subgroup analysis; **(G)** the results of publication bias.

#### Blurred vision incidence

Five studies documented the incidence of blurred vision (Hu, 2011; Liang, 2011; Cai, 2013; Cai et al., 2014; Ou, 2014). The *I* ^*2*^ statistic for the model was 0.00%, prompting the selection of a fixed-effect model for analysis. The findings indicated that the incidence of blurred vision following the injection of phloroglucinol was lower than that observed after the administration of anisodamine (Figure 6B).

#### Palpitations incidence

Five studies reported the incidence of palpitations (Hu, 2011; Liang, 2011; Qiu and Luo, 2011; Cai, 2013; Cai et al., 2014). With an *I* ^*2*^ value of 0.00%, a fixed-effect model was deemed appropriate for analysis. The results demonstrated that the incidence of palpitations was lower after the injection of phloroglucinol compared to after the administration of anisodamine (Figure 6C).

#### Meta regression, subgroup analysis, sensitivity analysis, and publication bias

Only the studies that reported the 1-h pain relief rate were sufficiently numerous to facilitate meta-regression and subgroup analyses. Consequently, based on the characteristics of the studies, we employed the doses of phloroglucinol and anisodamine as independent variables, with the 1-h pain relief rate serving as the dependent variable, to perform a meta-regression analysis. The findings revealed that the dose of phloroglucinol exerted a statistically significant impact on the outcome, indicating that phloroglucinol dosage was a source of heterogeneity (Figure 6E). We subsequently categorized the included studies into two subgroups based on phloroglucinol doses of 40–60 mg and 80 mg. The analysis demonstrated that the 1-h pain relief rate associated with phloroglucinol was superior to that of anisodamine, aligning with the results of the network meta-analysis and suggesting the robustness of the outcome. Furthermore, a sensitivity analysis using the leave-one-out method was conducted. Excluding each study sequentially, the 1-h pain relief rate for phloroglucinol consistently exceeded that of anisodamine, with no change in direction, further confirming the stability of the results (Figure 6D). Furthermore, in the revised funnel plot, the convergence lines of the ten studies aligned with the vertical axis formed an angle approaching 90°, indicating that the data points on either side of the vertical line were approximately symmetrical. This symmetry suggested the presence of a minor publication bias (Figure 6G).

## Discussion

As previously described, the development of nephrolithiasis represents the predominant cause of renal colic during pregnancy, which is largely attributable to the unique physiological alterations inherent to gestation. Within the framework of conservative management, antispasmodic therapy serves as a viable alternative for pain relief when conventional analgesics are contraindicated or provide suboptimal efficacy. In the present network meta-analysis, phloroglucinol demonstrated the highest efficacy in alleviating pain within 1 h, surpassing both progesterone and anisodamine. Furthermore, in the paired meta-analysis, phloroglucinol exhibited a lower 6-h pain recurrence rate compared to anisodamine. These findings are consistent with previous clinical research that has validated the effectiveness of phloroglucinol in alleviating renal colic (Corvino et al., 2023).

Phloroglucinol, along with its methylated derivative 1,3,5-trimethoxybenzene, is registered as an effective treatment for renal and gallbladder colic (Boubaker et al., 2010; Blanchard et al., 2018), with clinical studies confirming their potent antispasmodic properties (Corvino et al., 2023). Mechanistically, these compounds inhibit catechol-O-methyltransferase (COMT) enzymes, which play a crucial role in the metabolic breakdown of catecholamines in smooth muscle cells. By inhibiting this metabolic pathway, there is an increase in sympathetic tension within smooth muscle tissues, resulting in an antispasmodic effect (Liu and Hu, 2007). Interestingly, phloroglucinol exhibits greater efficacy on spastic muscles compared to normal muscles, indicating a higher selectivity in alleviating spasms and pain (Corvino et al., 2023).

Progesterone is a pharmacological agent classified as a hormone. Its analgesic mechanism is intricately associated with the modulation of ureteral contraction. Mechanistically, progesterone exerts its effects by inhibiting calcium ion channels, thereby diminishing the influx of extracellular Ca^2+^ into cells and reducing the release of intracellular calcium stores (Al-Shboul et al., 2018). This leads to a decrease in intracellular Ca^2+^ concentration within smooth muscle cells, resulting in smooth muscle relaxation (Nuss et al., 2005). Concurrently, progesterone mitigates the effects of prostaglandins, which are implicated in ureteral spasms, thereby reducing the excitability of ureteral smooth muscle (Bharadwaj et al., 2015; Piriyev et al., 2025). Furthermore, progesterone exerts an inhibitory influence on sympathetic nervous system activity, attenuating sensory impulses from sympathetic nerve afferent fibers to the ureter and thus achieving an analgesic effect (Wirth, 2011; Bencker et al., 2025). Furthermore, due to its structural similarity to aldosterone, progesterone can competitively inhibit aldosterone’s action in the renal tubules, thereby promoting the excretion of sodium and chloride ions (Quinkler et al., 2002; Arroyo et al., 2011). This results in a diuretic effect, characterized by increased urine flow, which aids in the expulsion of renal calculi and facilitates their movement. Additionally, progesterone has the capacity to relax the smooth muscle of the ureter and dilate the smooth muscle of the uterus (Pang and Thomas, 2018; Alqudah et al., 2022; Priyanto et al., 2022). Consequently, while managing renal colic, progesterone also exhibits an anti-fetal effect by reducing the risk of uterine contractions induced by severe pain, which could otherwise lead to miscarriage or premature birth. It is important to note, however, that pregnant patients already exhibit elevated levels of progesterone, which may diminish the spasmolytic efficacy of the hormone when administered therapeutically (Lee et al., 2014; Vincent et al., 2018; Kolatorova et al., 2022). As a result, many patients experiencing severe pain require adjunctive analgesic medications after progesterone administration to achieve adequate pain relief (Kolatorova et al., 2022). This limitation may contribute to the observation that progesterone’s analgesic efficacy is inferior to that of phloroglucinol.

Anisodamine acts as an M cholinergic receptor antagonist, effectively inhibiting the binding of acetylcholine to these receptors, which results in the relaxation of smooth muscle contractions (Poupko et al., 2007). Furthermore, anisodamine impedes the influx of calcium ions (Ca^2+^) into smooth muscle cells (Branch, 2025). Calcium ions serve as crucial messengers in the muscle contraction process; thus, a reduction in their concentration directly impairs the ability of smooth muscle fibers to contract efficiently, facilitating a transition from a contracted to a relaxed state (Branch, 2025). However, anisodamine is a broad-spectrum M cholinergic receptor antagonist, with a wide range of action (Zhang et al., 2023). While it is effective in alleviating ureteral spasms, it may also impact M receptors in other bodily regions, such as the salivary glands, heart, eyes, and intestines, potentially leading to side effects including xerostomia, tachycardia, blurred vision, and urinary retention (Branch, 2025). In pregnant women, these adverse effects may exacerbate discomfort, which leads to the discontinuation of therapy before a therapeutically effective antispasmodic dosage is reached. This may account for the comparatively reduced analgesic efficacy of anisodamine relative to phentolamine and progesterone.

For pregnant women, the safety of medications is a paramount concern, surpassing even the need for pain relief. This study demonstrates that phloroglucinol presents a lower incidence of adverse effects such as nausea and vomiting, dry mouth, flushing and hot flashes, urinary difficulties, dizziness, and headache compared to progesterone and anisodamine. Furthermore, phloroglucinol is associated with a reduced likelihood of causing blurred vision and palpitations relative to anisodamine. As previously noted, phloroglucinol exhibits a highly selective “myotropic” effect without anticholinergic properties, thereby avoiding common side effects such as dry mouth, flushing, and hot flashes, increased heart rate, blurred vision, and urinary difficulties, which are of particular concern with anisodamine use (Corvino et al., 2023; Wang et al., 2024). As for progesterone, it can induce smooth muscle spasms and systemic hormonal-like reactions, including nausea and dizziness (Kolatorova et al., 2022). These factors partially account for the findings of this study.

It is important to acknowledge that the investigation of side effects in this study was confined to short-term drug effects. The long-term effects of these drugs on pregnancy and fetal development were not considered, despite their significance for the clinical treatment of renal colic in pregnant women. This represents a limitation of the present study. Pioneer clinical research on the use of phloroglucinol in treating renal colic has not identified any serious adverse reactions (Corvino et al., 2023). Furthermore, a meta-analysis indicates that continuous low-dose administration before and after frozen embryo transfer in patients with endometriosis significantly increases clinical pregnancy and live birth rates (Yuan et al., 2019; Pang et al., 2022). No adverse pregnancy outcomes or fetal developmental abnormalities are observed in these cases, which provides support for the safety of phloroglucinol administration during pregnancy.

Regarding progesterone, beyond its roles in alleviating pain and preventing uterine contractions, it also enhances maternal immune tolerance to the fetus, thereby reducing immune system attacks on the fetus and supporting pregnancy continuation (Solano and Arck, 2019). During early pregnancy, progesterone can also facilitate the secretion and transformation of the endometrium, creating a suitable environment for embryo implantation (Saunders et al., 2020). Moreover, research has demonstrated a significant correlation between fluctuations in progesterone levels and the expression of antioxidant genes within the placenta, a relationship that is vital for the normal development of the fetus (Qi et al., 2020). Additionally, a systematic review demonstrates that prenatal exposure does not elevate the risk of impaired neurodevelopment in children, as no significant differences are found in Bayley-III cognitive scores (Simons et al., 2021). It is suggested that progesterone may even offer neuroprotective potential for fetuses with congenital heart disease, and long-term maternal assessments report no significant increase in adverse events (Gaynor et al., 2024). Therefore, although its analgesic efficacy may not match that of phloroglucinol, progesterone can be considered from a safety standpoint or considered as a potential candidate for combination therapy with phloroglucinol.

Conversely, human clinical data on the long-term fetal and maternal safety of anisodamine remain extremely limited. Basic research involving zebrafish embryos reveals that anisodamine influences pigmentation, mineral density, and the development of the cranial region and eyes, while high dosages lead to distinct developmental malformations (Wang et al., 2022). Additionally, anisodamine has shown cardioprotective properties in myocardial ischemia/reperfusion injury models by mitigating oxidative stress and inflammatory responses (Yao et al., 2018). However, it is important to recognize that excessive anti-inflammatory effects could potentially disrupt the normal immune system development in the fetus. Furthermore, anisodamine mediates its anti-shock effects through the activation of the α7 nicotinic acetylcholine receptor. This mechanism implies potential impacts on fetal neural development, given the critical role of the α7 nicotinic acetylcholine receptor in the development of the nervous system (Li et al., 2011; Sun et al., 2012). Consequently, considering the potential impact on long-term fetal development, anisodamine is less suitable than phloroglucinol and progesterone for the treatment of pregnancy-related renal colic. Although the present study does not yield such direct results, this descriptive conclusion is established through a review of existing literature.

Another limitation is the limited number of studies included in this analysis, which are generally of low methodological quality characterized by small sample sizes and single-center designs. The overall risk of bias is assessed as having some concerns, particularly regarding performance and detection bias, which arises primarily from the lack of blinding and the reliance on subjective outcome measures in the original trials. Recent research suggests that the absence of blinding for outcome assessors may moderately overestimate treatment effects, and the lack of blinding for patients or personnel can also lead to a slight overestimation when subjective outcomes are evaluated (Pitre et al., 2023). Nevertheless, despite these potential overestimations, the sensitivity and subgroup analyses indicate that the direction of the results remains stable and is not altered by any individual study. Furthermore, all included studies utilize active controls rather than placebos, which helps to offset a portion of the shared expectation bias. These factors likely mitigate the overestimation inherent in the research design, and readers should interpret the findings with these considerations in mind.

Furthermore, the missing of certain data in the primary studies, such as maternal age distribution and gestational age, restricts the ability to provide a comprehensive characterization of the research profile for the meta-analysis. Additionally, although a systematic search is performed across both Chinese and English databases, all included original studies are conducted in mainland China, which limits the generalizability of the findings. Pharmacological responses vary among different regions and ethnic groups, and therefore future research should validate the conclusions of this study in diverse geographic locations and populations (Olafuyi et al., 2021). Also, there exists some heterogeneity within the study; although we have partially addressed this issue through sensitivity analysis, meta-regression, and subgroup analysis, it remains a factor to consider when interpreting the results. On the other hand, although the findings indicate that the analgesic efficacy of phloroglucinol is superior to that of progesterone and anisodamine with a comparable safety profile, the optimal dosage and administration timing for this medication remain unexplored within the present study. This lack of granular data represents an additional limitation. Clinicians can consult the prescribing information provided by the manufacturer for specific guidance on clinical application.

Despite these limitations, the findings of this study provide valuable insights into antispasmodic analgesic treatment strategies for renal colic during pregnancy, particularly in cases where conventional analgesics are contraindicated or offer suboptimal efficacy.

## Conclusion

In pregnant women experiencing renal colic, phloroglucinol demonstrates a highly effective analgesic response and is associated with the lowest incidence of short-term adverse effects. Regarding long-term safety, pioneer research indicates that both phloroglucinol and progesterone may be viable options. However, these conclusions require further validation through multicenter studies with larger sample sizes.

## Data Availability

The original contributions presented in the study are included in the article/Supplementary Material, further inquiries can be directed to the corresponding authors.
