# Supplementary material for: Antispasmodic and analgesic strategies for renal colic in pregnancy: a comprehensive analysis
Source: Front Pharmacol. 2026 Oct 1;17:1758430. doi: 10.3389/fphar.2026.1758430 (PMC13668051; doi:10.3389/fphar.2026.1758430)

File S1: PRISMA checklist for network meta-analysis.

Section/topic	#	Checklist item
**TITLE**		
Title	1	Identify the report as a systematic review incorporating a network meta-analysis (or related form of meta-analysis).
**ABSTRACT**		
Structured summary	2	Provide a structured summary including, as applicable:<br>Background: main objectives<br>Methods: data sources; study eligibility criteria, participants, and interventions; study appraisal; and synthesis methods, such as network meta-analysis.<br>Results: number of studies and participants identified; summary estimates with corresponding confidence/credible intervals; treatment rankings may also be discussed. Authors may choose to summarize pairwise comparisons against a chosen treatment included in their analyses for brevity.<br>Discussion/Conclusions: limitations; conclusions and implications of findings.<br>Other: primary source of funding; systematic review registration number with registry name.
**INTRODUCTION**		
Rationale	3	Describe the rationale for the review in the context of what is already known, including mention of why a network meta-analysis has been conducted
Objectives	4	Provide an explicit statement of questions being addressed with reference to participants, interventions, comparisons, outcomes, and study design (PICOS).
**METHODS**		
Protocol and registration	5	Indicate if a review protocol exists and if and where it can be accessed (e.g., Web address), and, if available, provide registration information including registration number.

Eligibility criteria	6	Specify study characteristics (e.g., PICOS, length of follow-up) and report characteristics (e.g., years considered, language, publication status) used as criteria for eligibility, giving rationale. Clearly describe eligible treatments included in the treatment network and note whether any have been clustered or merged into the same node (with justification).
Information sources	7	Describe all information sources (e.g., databases with dates of coverage, contact with study authors to identify additional studies) in the search and date last searched.
Search	8	Present full electronic search strategy for at least one database, including any limits used, such that it could be repeated.
Study selection	9	State the process for selecting studies (i.e., screening, eligibility, included in systematic review, and, if applicable, included in the meta-analysis).
Data collection process	10	Describe method of data extraction from reports (e.g., piloted forms, independently, in duplicate) and any processes for obtaining and confirming data from investigators.
Data items	11	List and define all variables for which data were sought (e.g., PICOS, funding sources) and any assumptions and simplifications made.
Geometry of the network	S1	Describe methods used to explore the geometry of the treatment network under study and potential biases related to it. This should include how the evidence base has been graphically summarized for presentation, and what characteristics were compiled and used to describe the evidence base to readers
Risk of bias within individual studies	12	Describe methods used for assessing risk of bias of individual studies (including specification of whether this was done at the study or outcome level), and how this information is to be used in any data synthesis.
Summary measures	13	State the principal summary measures (e.g., risk ratio, difference in means). Also describe the use of additional summary measures assessed, such as treatment rankings and surface under the cumulative ranking curve (SUCRA) values, as well as modified approaches used to present summary findings from meta-analyses.
Planned methods of analysis	14	Describe the methods of handling data and combining results of studies for each network meta-analysis. This should include, but not be limited to: Handling of multigroup trials; Selection of variance structure; Selection of prior distributions in Bayesian analyses; and Assessment of model fit.
Assessment of inconsistency	S2	Describe the statistical methods used to evaluate the agreement of direct and indirect evidence in the treatment network(s) studied. Describe efforts taken to address its presence when found.
Risk of bias across studies	15	Specify any assessment of risk of bias that may affect the cumulative evidence (e.g., publication bias, selective reporting within studies)
Additional analyses	16	Describe methods of additional analyses if done, indicating which were prespecified. This may include, but not be limited to, the following: Sensitivity or subgroup analyses; Meta-regression analyses; Alternative formulations of the treatment network; and Use of alternative prior distributions for Bayesian analyses (if applicable).
**RESULTS**		

Study selection	17	Give numbers of studies screened, assessed for eligibility, and included in the review, with reasons for exclusions at each stage, ideally with a flow diagram.
Presentation of network structure	S3	Provide a network graph of the included studies to enable visualization of the geometry of the treatment network.
Summary of network geometry	S4	Provide a brief overview of characteristics of the treatment network. This may include commentary on the abundance of trials and randomized patients for the different interventions and pairwise comparisons in the network, gaps of evidence in the treatment network, and potential biases reflected by the network structure.
Study characteristics	18	For each study, present characteristics for which data were extracted (e.g., study size, PICOS, follow-up period) and provide the citations.
Risk of bias within studies	19	Present data on risk of bias of each study and, if available, any outcome level assessment.
Results of individual studies	20	For all outcomes considered (benefits or harms), present, for each study: 1) simple summary data for each intervention group, and 2) effect estimates and confidence intervals. Modified approaches may be needed to deal with information from larger networks.
Synthesis of results	21	Present results of each meta-analysis done, including confidence/credible intervals. In larger networks, authors may focus on comparisons versus a particular comparator (e.g., placebo or standard care), with full findings presented in an appendix. League tables and forest plots may be considered to summarize pairwise comparisons. If additional summary measures were explored (such as treatment rankings), these should also be presented.
Exploration for inconsistency	S5	Describe results from investigations of inconsistency. This may include such information as measures of model fit to compare consistency and inconsistency models, P values from statistical tests, or summary of inconsistency estimates from different parts of the treatment network.
Risk of bias across studies	22	Present results of any assessment of risk of bias across studies for the evidence base being studied.
Results of additional analyses	23	Give results of additional analyses, if done (e.g., sensitivity or subgroup analyses, meta-regression analyses, alternative network geometries studied, alternative choice of prior distributions for Bayesian analyses, and so forth).
**DISCUSSION**		
Summary of evidence	24	Summarize the main findings, including the strength of evidence for each main outcome; consider their relevance to key groups (e.g., health care providers, researchers, and policymakers).
Limitations	25	Discuss limitations at study and outcome level (e.g., risk of bias), and at review level (e.g., incomplete retrieval of identified research, reporting bias). Comment on the validity of the assumptions, such as transitivity and consistency. Comment on any concerns regarding network geometry (e.g., avoidance of certain comparisons).

Conclusions	26	Provide a general interpretation of the results in the context of other evidence, and implications for future research.
**FUNDING**		
Funding	27	Describe sources of funding for the systematic review and other support (e.g., supply of data); role of funders for the systematic review. This should also include information regarding whether funding has been received from manufacturers of treatments in the network and/or whether some of the authors are content experts with professional conflicts of interest that could affect use of treatments in the network.

File S2: Search strategy for network meta-analysis.

Search strategy of China National Knowledge Infrastructure.

No.	Search items
#1	SU %= '肾绞痛' OR SU %= '急性肾绞痛' OR SU %= '泌尿系结石' OR SU %= '泌尿道结石' OR SU %= '泌尿系疾病' OR SU %= '输尿管绞痛' OR SU %= '肾结石' OR SU %= '输尿管结石' OR SU %= '膀胱结石'
#2	SU %= '间苯三酚' OR SU %= '斯帕丰' OR SU %= '山莨菪碱' OR SU %= '654-2' OR SU %= '黄体酮'
#3	SU %= '孕妇' OR SU %= '妊娠' OR SU %= '孕期' OR SU %= '妊娠期' OR SU %= '怀孕'
#4	FT = '随机'
#5	#1 AND #2 AND #3 AND #4

Search strategy of Wanfang Database.

No.	Search items
#1	主题:(肾绞痛) or 主题:(急性肾绞痛) or 主题:(泌尿系结石) or 主题:(泌尿道结石) or 主题:(泌尿系疾病) or 主题:(输尿管绞痛) or 主题:(肾结石) or 主题:(输尿管结石) or 主题:(膀胱结石)

#2	主题:(孕妇) or 主题:(妊娠) or 主题:(孕期) or 主题:(妊娠期) or 主题:(怀孕)
#3	主题:(间苯三酚) or 主题:(斯帕丰)) or (主题:(山莨菪碱) or 主题:(654-2) or 主题:(黄体酮)
#4	全部:(随机)
#5	#1 and #2 and #3 and #4

Search strategy of Chinese Biomedical Literature Database.

No.	Search items
#1	"肾绞痛"[常用字段:智能] OR "泌尿系结石"[常用字段:智能] OR "泌尿道结石"[常用字段:智能] OR "泌尿系疾病"[常用字段:智能] OR "肾结石"[常用字段:智能] OR "输尿管结石"[常用字段:智能] OR "膀胱结石"[常用字段:智能]
#2	"孕妇"[常用字段:智能] OR "妊娠"[常用字段:智能] OR "怀孕"[常用字段:智能] OR "妊娠期"[常用字段:智能] OR "孕期"[常用字段:智能]
#3	"山莨菪碱"[常用字段:智能] OR "654-2"[常用字段:智能] OR "间苯三酚"[常用字段:智能] OR "斯帕丰"[常用字段:智能] OR "黄体酮"[常用字段:智能]
#4	"随机"[全部字段:智能])

#5	#1 AND #2 AND #3 AND #4

Search strategy of Weipu Journal Database.

No.	Search items
#1	M=(肾绞痛 OR 泌尿系结石 OR 泌尿道结石 OR 泌尿系疾病 OR 肾结石 OR 输尿管结石 OR 膀胱结石)
#2	M=(孕妇 OR 孕期 OR 妊娠 OR 妊娠期 OR 怀孕)
#3	M=(间苯三酚 OR 山莨菪碱 OR 654-2 OR 黄体酮)
#3	U=(随机)
#4	#1 AND #2 AND #3

Search strategy of Pubmed.

No.	Search items

#1	("Randomized Controlled Trial" [Publication Type]) OR (((((Controlled Clinical Trial[Publication Type]) OR (Equivalence Trial[Publication Type])) OR (Pragmatic Clinical Trial[Publication Type])) OR (Equivalence Trial[Publication Type])) OR (random*))
#2	("anisodamine" [Supplementary Concept]) OR (((anisodamine hydrobromide[Title/Abstract]) OR (racanisodamine[Title/Abstract])) OR (6-hydroxyhyoscyamine[Title/Abstract])) OR ("Phloroglucinol"[Mesh]) OR ((Benzene-1,3,5-triol[Title/Abstract]) OR (1,3,5-Benzenetriol[Title/Abstract])) OR (progesterone[Title/Abstract])
#3	(((("Pregnant Women"[Mesh]) OR (((Pregnant Woman[Title/Abstract]) OR (Woman, Pregnant[Title/Abstract])) OR (Women, Pregnant[Title/Abstract]))) OR ("Pregnancy"[Mesh])) OR ((Pregnancies[Title/Abstract]) OR (Gestation[Title/Abstract]))) OR ("Gravidity"[Mesh])
#4	((((((((((((((((((((((("Renal Colic"[Mesh]) OR (((((((((((((Colic, Renal[Title/Abstract]) OR (Colics, Renal[Title/Abstract])) OR (Renal Colics[Title/Abstract])) OR (Acute Renal Colic[Title/Abstract])) OR (Acute Renal Colics[Title/Abstract])) OR (Colic, Acute Renal[Title/Abstract])) OR (Colics, Acute Renal[Title/Abstract])) OR (Renal Colic, Acute[Title/Abstract])) OR (Renal Colics, Acute[Title/Abstract])) OR (Ureteral Colic[Title/Abstract])) OR (Colic, Ureteral[Title/Abstract])) OR (Colics, Ureteral[Title/Abstract])) OR (Ureteral Colics[Title/Abstract]))) OR (kidney pain[Title/Abstract])) OR ("Kidney Calculi"[Mesh])) OR ((((((((((((Calculi, Kidney[Title/Abstract]) OR (Calculus, Kidney[Title/Abstract])) OR (Kidney Calculus[Title/Abstract])) OR (Nephrolith[Title/Abstract])) OR (Renal Calculus[Title/Abstract])) OR (Kidney Stones[Title/Abstract])) OR (Kidney Stone[Title/Abstract])) OR (Stone, Kidney[Title/Abstract])) OR (Stones, Kidney[Title/Abstract])) OR (Renal Calculi[Title/Abstract])) OR (Calculi, Renal[Title/Abstract])) OR (Calculus, Renal[Title/Abstract]))) OR ("Ureteral Calculi"[Mesh])) OR (((Calculi,

Ureteral[Title/Abstract]) OR (Calculus, Ureteral[Title/Abstract])) OR (Ureteral Calculus[Title/Abstract]))) OR ("Urinary Bladder Calculi"[Mesh])) OR ((((((((((((((((((((((((((Bladder Calculi, Urinary[Title/Abstract]) OR (Bladder Calculus, Urinary[Title/Abstract])) OR (Urinary Bladder Calculus[Title/Abstract])) OR (Calculi, Urinary Bladder[Title/Abstract])) OR (Calculus, Urinary Bladder[Title/Abstract])) OR (Bladder Stones[Title/Abstract])) OR (Bladder Stone[Title/Abstract])) OR (Stone, Bladder[Title/Abstract])) OR (Stones, Bladder[Title/Abstract])) OR (Vesical Calculi[Title/Abstract])) OR (Calculi, Vesical[Title/Abstract])) OR (Calculus, Vesical[Title/Abstract])) OR (Vesical Calculus[Title/Abstract])) OR (Cystoliths[Title/Abstract])) OR (Cystolith[Title/Abstract])) OR (Urinary Bladder Stones[Title/Abstract])) OR (Bladder Stone, Urinary[Title/Abstract])) OR (Bladder Stones, Urinary[Title/Abstract])) OR (Stone, Urinary Bladder[Title/Abstract])) OR (Stones, Urinary Bladder[Title/Abstract])) OR (Urinary Bladder Stone[Title/Abstract])) OR (Bladder Calculi[Title/Abstract])) OR (Bladder Calculus[Title/Abstract])) OR (Calculi, Bladder[Title/Abstract])) OR (Calculus, Bladder[Title/Abstract])) OR (Calculi of Urinary Bladder[Title/Abstract]))) OR (ureteral stones[Title/Abstract])) OR ("Urinary Calculi"[Mesh])) OR (((((((((((Calculi, Urinary[Title/Abstract]) OR (Calculus, Urinary[Title/Abstract])) OR (Urinary Calculus[Title/Abstract])) OR (Urinary Tract Stones[Title/Abstract])) OR (Stone, Urinary Tract[Title/Abstract])) OR (Stones, Urinary Tract[Title/Abstract])) OR (Urinary Tract Stone[Title/Abstract])) OR (Urinary Stones[Title/Abstract])) OR (Stone, Urinary[Title/Abstract])) OR (Stones, Urinary[Title/Abstract])) OR (Urinary Stone[Title/Abstract]))) OR ("Urolithiasis"[Mesh])) OR ((Urinary Lithiasis[Title/Abstract]) OR (Lithiasis, Urinary[Title/Abstract]))) OR ("Nephrolithiasis"[Mesh])) OR ("Ureteral Diseases"[Mesh])) OR (((Disease, Ureteral[Title/Abstract]) OR (Diseases, Ureteral[Title/Abstract])) OR (Ureteral Disease[Title/Abstract]))) OR ("Ureteral Obstruction"[Mesh])) OR (((Obstruction, Ureteral[Title/Abstract]) OR (Obstructions, Ureteral[Title/Abstract])) OR (Ureteral Obstructions[Title/Abstract]))) OR ("Kidney Diseases"[Mesh])) OR (((Disease, Kidney[Title/Abstract]) OR (Diseases, Kidney[Title/Abstract])) OR (Kidney Disease[Title/Abstract]))) OR ("Urologic Diseases"[Mesh])) OR (((((((((((Disease, Urologic[Title/Abstract]) OR (Diseases,

	Urologic[Title/Abstract])) OR (Urologic Disease[Title/Abstract])) OR (Urological Diseases[Title/Abstract])) OR (Disease, Urological[Title/Abstract])) OR (Diseases, Urological[Title/Abstract])) OR (Urological Disease[Title/Abstract])) OR (Urinary Tract Diseases[Title/Abstract])) OR (Disease, Urinary Tract[Title/Abstract])) OR (Diseases, Urinary Tract[Title/Abstract])) OR (Urinary Tract Disease[Title/Abstract]))
#5	#1 AND #2 AND #3

Search strategy of Embase.

No.	Search items
#1	'kidney colic'/exp OR 'colic, kidney':ab,ti OR 'colic, ureteral':ab,ti OR 'renal colic':ab,ti OR 'ureteral colic':ab,ti OR 'nephrolithiasis'/exp OR 'calculosis, kidney':ab,ti OR 'calculus, kidney':ab,ti OR 'familial nephrolithiasis':ab,ti OR 'kidney calculi':ab,ti OR 'kidney calculosis':ab,ti OR 'kidney calculus':ab,ti OR 'kidney calix stone':ab,ti OR 'kidney calyx stone':ab,ti OR 'kidney lithiasis':ab,ti OR 'kidney pelvis stone':ab,ti OR 'kidney stone':ab,ti OR 'kidney stone passage':ab,ti OR 'kidney stone, pelvis':ab,ti OR 'kidney stones':ab,ti OR nephrolith:ab,ti OR 'nephrolith passage':ab,ti OR 'renal calculogenesis':ab,ti OR 'renal calculosis':ab,ti OR 'renal calculus':ab,ti OR 'renal lithiasis':ab,ti OR 'renal pelvis stone':ab,ti OR 'renal stone':ab,ti OR renolithiasis:ab,ti OR 'stone, kidney':ab,ti OR 'ureter stone'/exp OR 'calculosis, ureter':ab,ti OR 'calculus, ureter':ab,ti OR 'stone, ureter':ab,ti OR 'ureter calculus':ab,ti OR 'ureter lithiasis':ab,ti OR 'ureteral calculi':ab,ti OR 'ureteral calculus':ab,ti OR 'ureteral stone':ab,ti OR 'ureteric calculus':ab,ti OR ureterolithiasis:ab,ti OR 'bladder stone'/exp OR 'bladder calculi':ab,ti OR 'bladder calculosis':ab,ti OR 'bladder calculus':ab,ti OR 'bladder concrement':ab,ti OR 'bladder concretion':ab,ti OR 'bladder lithiasis':ab,ti OR 'bladder neck calculus':ab,ti OR 'bladder neck stone':ab,ti OR 'bladder

	stone disease':ab,ti OR 'bladder stones':ab,ti OR 'bladder urolith':ab,ti OR 'bladder urolithiasis':ab,ti OR 'bladder uroliths':ab,ti OR (cystolithiasis:ab,ti AND bladder:ab,ti) OR 'urinary bladder calculi':ab,ti OR 'urinary bladder stone':ab,ti OR 'urinary bladder stones':ab,ti OR 'vesical calculi':ab,ti OR 'vesical calculus':ab,ti OR 'vesical lithiasis':ab,ti OR 'vesical stone':ab,ti OR 'vesical stones':ab,ti OR vesicolithiasis:ab,ti OR 'urolithiasis'/exp OR 'coral stone':ab,ti OR 'stone, urinary tract':ab,ti OR 'stone, urine':ab,ti OR 'urinary calculi':ab,ti OR 'urinary calculogenesis':ab,ti OR 'urinary calculoses':ab,ti OR 'urinary calculosis':ab,ti OR 'urinary calculus':ab,ti OR 'urinary lithiasis':ab,ti OR 'urinary stone':ab,ti OR 'urinary tract calculi':ab,ti OR 'urinary tract calculosis':ab,ti OR 'urinary tract calculus':ab,ti OR 'urinary tract lithiasis':ab,ti OR 'urinary tract stone':ab,ti OR 'urine calculi':ab,ti OR 'urine calculus':ab,ti OR 'urine lithiasis':ab,ti OR 'urine stone':ab,ti OR 'uro lithiasis':ab,ti OR urocalculi:ab,ti OR urocalculogenesis:ab,ti OR urocalculosis:ab,ti OR urolith:ab,ti OR urolithogenesis:ab,ti OR 'urologic calculi':ab,ti OR 'urological calculi':ab,ti OR urolyt:ab,ti OR 'urinary tract disease'/exp OR 'urinary tract disorder':ab,ti OR 'urine tract disease':ab,ti OR 'urologic disease':ab,ti OR 'urologic diseases':ab,ti OR 'urological disease':ab,ti OR 'urological manifestations':ab,ti OR uropathy:ab,ti OR 'ureter disease'/exp OR 'ureteral diseases':ab,ti OR ureteropathy:ab,ti OR 'kidney disease'/exp OR 'disease, kidney':ab,ti OR 'kidney diseases':ab,ti OR 'kidney disorder':ab,ti OR 'kidney pathology':ab,ti OR nephropathy:ab,ti OR perinephritis:ab,ti OR 'perirenal infection':ab,ti OR 'renal disease':ab,ti OR 'renal disorder':ab,ti OR 'unilateral kidney disease':ab,ti OR 'ureter obstruction'/exp OR 'distal ureter stenosis':ab,ti OR 'distal ureteral stenosis':ab,ti OR 'pyeloureteric stenosis':ab,ti OR 'ureter constriction':ab,ti OR 'ureter occlusion':ab,ti OR 'ureter ostium stenosis':ab,ti OR 'ureter stenosis':ab,ti OR 'ureteral obstruction':ab,ti OR 'ureteral occlusion':ab,ti OR 'ureteral stenosis':ab,ti OR 'ureteric obstruction':ab,ti OR ureterstenosis:ab,ti
#2	'pregnant woman'/exp OR 'pregnant women':ab,ti OR 'pregnancy'/exp OR 'child bearing':ab,ti OR childbearing:ab,ti OR gestation:ab,ti OR gravidity:ab,ti OR 'intrauterine pregnancy':ab,ti OR 'labor

	presentation':ab,ti OR 'labour presentation':ab,ti OR 'pregnancy maintenance':ab,ti OR 'pregnancy trimesters':ab,ti
#3	'phloroglucinol'/exp OR '1, 3, 5 benzenetriol':ab,ti OR '1, 3, 5 trihydroxybenzene':ab,ti OR '3, 5 dihydroxyphenol':ab,ti OR 'crl 572':ab,ti OR crl572:ab,ti OR floroglucin:ab,ti OR 'floroglucin phosphate':ab,ti OR floroglucinol:ab,ti OR phlorochlucine:ab,ti OR phloroglucin:ab,ti OR 'phloroglucinol hydrate':ab,ti OR 'spasfon lyoc':ab,ti OR 'anisodamine'/exp OR '6 hydroxyhyoscyamine':ab,ti OR 'hyoscyamine, 6 hydroxy':ab,ti OR 'progesterone':ab,ti
#4	'randomized controlled trial'/exp OR 'equivalence trial'/exp OR 'non-inferiority trial'/exp OR 'pragmatic trial'/exp OR 'superiority trial'/exp OR 'controlled clinical trial':it OR 'randomized controlled trial':it OR 'equivalence trial':it OR 'pragmatic clinical trial':it OR 'superiority trial':it OR 'non-inferiority trial':it OR random*
#5	#1 AND #2 AND #3 AND #4

Search strategy of Cochrane Library.

No.	Search items
#1	MeSH descriptor: [Renal Colic] explode all trees OR (Renal Colic, Acute):ti,ab,kw OR (Ureteral Colics):ti,ab,kw OR (Acute Renal Colics):ti,ab,kw OR (Renal Colics):ti,ab,kw OR (Renal Colics, Acute):ti,ab,kw OR (Colics, Acute Renal):ti,ab,kw OR (Colics, Renal):ti,ab,kw OR (Colics, Ureteral):ti,ab,kw OR (Ureteral Colic):ti,ab,kw OR (Acute Renal Colic):ti,ab,kw OR (Colic, Acute

Renal):ti,ab,kw OR (Colic, Ureteral):ti,ab,kw OR (Colic, Renal):ti,ab,kw OR (kidney pain):ti,ab,kw OR MeSH descriptor: [Nephrolithiasis] explode all trees OR (Kidney Calculus):ti,ab,kw OR (KIDNEY CALCULI):ti,ab,kw OR (Kidney Stones):ti,ab,kw OR (Renal Calculus):ti,ab,kw OR (Calculi, Kidney):ti,ab,kw OR (Renal Calculi):ti,ab,kw OR (Stone, Kidney):ti,ab,kw OR (Calculus, Renal):ti,ab,kw OR (Kidney Stone):ti,ab,kw OR (Stones, Kidney):ti,ab,kw OR (Calculi, Renal):ti,ab,kw OR (Calculus, Kidney):ti,ab,kw OR (Nephrolith):ti,ab,kw OR MeSH descriptor: [Ureteral Calculi] explode all trees OR (Calculus, Ureteral):ti,ab,kw OR (Ureteral Calculus):ti,ab,kw OR (Calculi, Ureteral):ti,ab,kw OR (Urinary Bladder Calculi):ti,ab,kw OR (Bladder stone):ti,ab,kw OR MeSH descriptor: [Urinary Calculi] explode all trees OR (Calculi, Urinary):ti,ab,kw OR (Urinary Stone):ti,ab,kw OR (Urinary Tract Stones):ti,ab,kw OR (Stones, Urinary):ti,ab,kw OR (Stones, Urinary Tract):ti,ab,kw OR (Urinary Tract Stone):ti,ab,kw OR (Urinary Calculus):ti,ab,kw OR (Calculus, Urinary):ti,ab,kw OR (Stone, Urinary Tract):ti,ab,kw OR (Urinary Stones):ti,ab,kw OR (Stone, Urinary):ti,ab,kw OR MeSH descriptor: [Urolithiasis] explode all trees OR (Urinary Lithiasis):ti,ab,kw OR (Lithiasis, Urinary):ti,ab,kw OR MeSH descriptor: [Ureteral Obstruction] explode all trees OR (Obstructions, Ureteral):ti,ab,kw OR (Obstruction, Ureteral):ti,ab,kw OR (Ureteral Obstructions):ti,ab,kw OR MeSH descriptor: [Urologic Diseases] explode all trees OR (Urological Diseases):ti,ab,kw OR (Diseases, Urologic):ti,ab,kw OR (Disease, Urologic):ti,ab,kw OR (Urinary Tract Disease):ti,ab,kw OR (Urologic Disease):ti,ab,kw OR (Diseases, Urological):ti,ab,kw OR (Disease, Urinary Tract):ti,ab,kw OR (Urological Disease):ti,ab,kw OR (Disease, Urological):ti,ab,kw OR (Diseases, Urinary Tract):ti,ab,kw OR (Urinary Tract Diseases):ti,ab,kw OR MeSH descriptor: [Ureteral Diseases] explode all trees OR (Diseases, Ureteral):ti,ab,kw OR (Ureteral Disease):ti,ab,kw OR (Disease, Ureteral):ti,ab,kw OR MeSH descriptor: [Kidney Diseases] explode all trees OR (Diseases, Kidney):ti,ab,kw OR (Kidney Disease):ti,ab,kw OR (Disease, Kidney):ti,ab,kw

#2	MeSH descriptor: [Pregnant Women] explode all trees OR (Pregnant Woman):ti,ab,kw OR (Woman, Pregnant):ti,ab,kw OR (Women, Pregnant):ti,ab,kw OR MeSH descriptor: [Pregnancy] explode all trees OR (Gestation):ti,ab,kw OR (Pregnancies):ti,ab,kw OR MeSH descriptor: [Gravidity] explode all trees
#3	(Phloroglucinol):ti,ab,kw OR ("anisodamine"):ti,ab,kw OR (6 hydroxyhyoscyamine):ti,ab,kw OR (hyoscyamine, 6 hydroxy):ti,ab,kw OR (progesterone):ti,ab,kw
#4	(Controlled Clinical Trial):pt OR (Randomized Controlled Trial):pt OR (Pragmatic Clinical Trial):pt OR (Equivalence Trial):pt OR (randomised controlled study):pt OR MeSH descriptor: [Randomized Controlled Trial] explode all trees
#5	#1 AND #2 AND #3 AND #4

File S3: Flow chart.

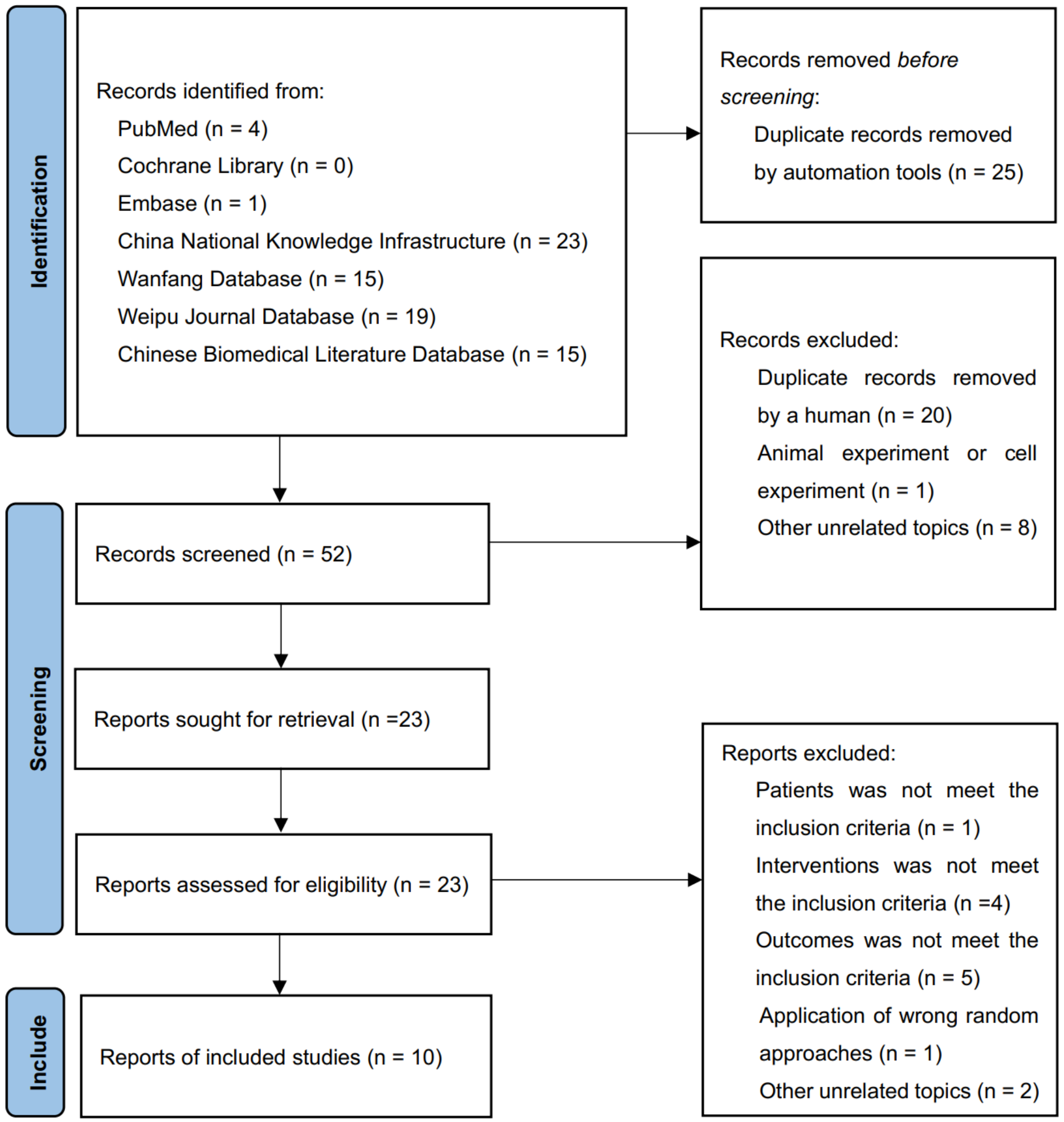

Supplement: Supplementary file 1 [file DataSheet1.pdf]
